# Supplementary material for: Genome-Wide Analysis of MADS-Box Gene Family Reveals CjSTK as a Key Regulator of Seed Abortion in Camellia japonica
Source: Int J Mol Sci. 2024 May 25;25(11):5770. doi: 10.3390/ijms25115770 (PMC11171818; doi:10.3390/ijms25115770)
Supplement: Supplementary file 1 [file ijms-25-05770-s001.zip › ijms-2990805-Supplementary Material.pdf]

Supplementary file:
sequences of C/D -class genes from various plant species
>AT4G18960_AG
TAYQSELGGDSSPLRKSGRGKIEIKRIENTTNRQVTFCKRRNGLLKKAYELSVLCDAEVALI
VFSSRGRLYEYSNNSVKGTIERYKKAISDNSNTGSVAEINAQYYQQESAKLRQQIISIQNSN
RQLMGETIGSMSPKELRNLEGRLERSITRIRSKKNELLFSEIDYMQKREVDLHNDNQILRA
KIAENERNNPSISLMPGGSNYEQLMPPPQTQSQPFDSRNYFQVAALQPNNHHYSSAGRQD
QTALQLV

>Ach19g182651.2-TA-protein
MGGRGKIEIKRIENTTNRQVTFCKRRNGLLKKAYELSVLCDAEVALIVFSTRGRLYEYANN
SVKGTIERYKKASSGSSNTGSVSELNAQFYQQEAAKLRVQINNLQSSNSHMRGESLSSLSL
RELKNLEGRLERGISRIRSKKIAEGERVQQMNLMPGGSEYELMQPPSFDTRNYLQVDGRL
QSDHNSYSRQDQTVLQLV

>TAG1
SVLCDAEVALVVFSNRGRLYEYANNSVKATIERYKKACSDSSNTGSVSEANAQYYQQEAS
KLRAQIGNLMNQNRNMMGEALAGMKLKELKNLEQRIEKGISKIRSKKNELLFAEIEYMQ
KREVDLHNNNQYLRAKIAETERAQHQHQQMNLMPGSSSNYHELVPPPQQFDTRNYLQV
NGLQTNNHYPRQDQPPIQLV

>EVM0034730.2_AG
MEFQNQSMDVSRLSPQRKMGRGKIEIKRIENTTNRQVTFCKRRNGLLKKAYELSVLCDAE
VALIVFSTRGRLYEYANNSVKATIERYKKACSGPSNTGSVSELNAQFYQQEAAKLRVQIGN
LQNSNRHMRGESLCSLSMRDLKNLEGRLERGISRIRSKKNELLFAEIELMQKREIDLHNNN
QYLRAKIAENERAQQQMSLMPAGPEYELMPSQSFDARNYLQMNGIQQQQPQQQQQQP
PNHHHYSRHDQTALQLV

>EVM0003627.3_AG2
MSYLNQSMEVLDSPQRKIGRGKIEIKRIENTTNRQVTFCKRRNGLLKKAYELSVLCDAEV
ALIVFSTRGRLYEYSNNSVKGTIERYKKACSDSSNTGSVSELNAQFYQQEAAKLRGQISNL
QNSHRQMLGESLSSMSIRDLKNLESRLERGISRIRSKKNELLFAEIELMQQREIDLHNSNQY
LRAKIAENERAQQQMNLMPGGGSEYELMPPPQSFDTRNYFQVNGLQPNDHYSRHDQTAL
QLV

>EVM0034505.1 gene=EVM0034505
MQPLWKMMVFPNQESEEGSSQRKMGRGKIEIKRIENTTNRQVTFCKRRNGLLKKAYELS
VLCDAEVALIVFSSRGRLYEYANNSVRATIERYKKACSDVPNTGSVSEANTQFYQQESTKL
RRQIKDIQNSNRHILGEALSSLTFKELKNLESRLEKAISRVRSKKNELLFAEIEHMQKREIEL
QNANMYLRAKIAENERAQQQEQEQEQMNLMPAGGGGGGGMGAYDARNFFPVNLLEPSP
PPPNHHYSCHDQTPLQLV

>Ach00g367721.2-TA-protein
MGRGKIEIKRIENTTNRQVTFCKRRNGLLKKAYELSVLCDAEVALIVFSSRGRLYEYANNS

VRTTIDRYKKACSDVLNTGSISESNTQFYQQESNKLRRQIKDIQSSNKHILGEALSSLTFKE
LKNLEGRLEKAISRIRSKKIAENERAQQQMNLMQGAEYQGMPSQQYDARNFLPVNLLEP
NQQYSRQDQTALQLV

>Ach00g243501.2-TA-protein
MGRGKIEIKRIENTTNRQVTFCKRRNGLLKKAYELSVLCDAEVALIVFSTRGRLYEYANNS
VRSTIDRYKKACSDVLNTGSVSQSNTQTPALGKFRAPSFVCYFPSSIAENERAQQQMNLM
PGAEYQDMPSQPYDARIFLPVNLLEPNHQYSRQDQTALHLV

>AT2G42830_SHP2
MEGGASNEVAESSKKIGRGKIEIKRIENTTNRQVTFCKRRNGLLKKAYELSVLCDAEVALV
IFSTRGRLYEYANNSVRGTIERYKKACSDAVNPPTITEANTQYYQQEASKLRRQIRDIQNLN
RHILGESLGSLNFKELKNLESRLEKGISRVRSKKHEMLVAEIEYMQKRVKEIELQNDNMYL
RSKITERTGLQQQESSVIHQGTVYESGVTSSHQSGQYNRNYIAVNLLEPNQNSSNQDQPPL
QLV

>AT3G58780_SHP1
MEEGGSSHDAESSKKLGRGKIEIKRIENTTNRQVTFCKRRNGLLKKAYELSVLCDAEVAL
VIFSTRGRLYEYANNSFIYLLLEKKKKKKKKNLWIYSSHVVRGTIERYKKACSDAVNPPS
VTEANTQYYQQEASKLRRQIRDIQNSNRHIVGESLGSLNFKELKNLEGRLEKGISRVRSKK
NELLVAEIEYMQKREMELQHNNMYLRAKIAEGARLNPDQQESSVIQGTTVYESGVSSHD
QSQHYNRNYIPVNLLEPNQQFSGQDQPPLQLV

>AtSTK
MGRGKIEIKRIENSTNRQVTFCKRRNGLLKKAYELSVLCDAEVALIVFSTRGRLYEYANNN
IRSTIERYKKACSDSTNTSTVQEINAAYYQQESAKLRQQIQTIQNSNRNLMGDSLSSLSVKE
LKQVENRLEKAISRIRSKKHELLLVEIENAQKREIELDNENIYLRTKVAEVERYQQHHHQM
VSGSEINAIEALASRNYFAHSIMTAGSGSGNGGSYSDPDKKILHLG

>EVM0019646.1
MGRGKIEIKRIENNTNRQVTFCKRRNGLLKKAYELSVLCEAEVALIVFSSRGRVYEYANNN
IKSTIERYKKAVADNSNPCPTPEINAQFYQQESKKLRQQIQMIQNTNRSLMGEGLDCLNMK
ELKQLENRLERGITRIRSKKHEMILAETENLQKREMELENENAFLRAKIAETERIQEQNMV
PGEEYNAIQAYFARNDLVLQLNIMDAEHPPPPPPAAYQRFDKKSLHLG

>Ach21g394681.2-TA-protein
MGRGKIEIKRIENNTNRQVTFCKRRNGLLKKAYELSVLCDAEVALIVFSSRGRVYEYANN
NIKSTIERYKKALADRSNACPTPEINTRELELEHENAILRSKIAENERLQELSMVSGQEYNA
IQAYLARNALQLNILEGGPGSYPPLPDKKSLHLG

>Ach00g421001.2-TA-protein
MGRGKIEIKKIENNTNRQVTFCKRRNGLLKKAYELSVLCDAEVALIVFSSRGRVYEYANN
NIKSTIERYKKAVADSSNACPPPEINARVDYDTWSFEVFI

>TAGL1
MVFPINQELLVDESSSQLRKTSGGTGGGGRGKIEIKRIENTTNRQVTFCKRRNGLLKKAYE
LSVLCDAEVSLIVFSSRGRLYEYANNSVRATIDRYKKHHADSTSTGSVSEANTQYYQQEAS
KLRRQIRDIQTYNRQIVGEALGSLSPRDLKNLEGKLEKAIGRVRSKKNELLFSEIELMQKR
EIELQNANMYLRAKIAEVERAQEQMNLMPGGGGGGGGGGGGGSDHQYHHQPNYEDAR
NNSLPVNLLEPNPHYSRRDNGDQTPLQLV

>AmFAR
MASLSDQSTEVSPERKIGRGKIEIKRIENKTNQQVTFCKRRNGLLKKAYELSVLCDAEVAL
VVFSSRGRLYEYANNSVKATIDRYKKASSDSSLNGSISEATQYYQQEASKLRAQISNLQNQ
NRNMLGESLGALSLRELKNLESRVERGISRIRSKKNELLFAEIEYMQKRQEIDLHHNNQYL
RAKIAESERVQGQHMNLMPGGSSGYEQLVETQPFDARNYLQVNGLQPNNDYPRQDQLPL
QLV

>Rhsim06G0119100
mafpsqdseg ssqrklgrgk ieikrientt nrqvtfckrr ngllkkayel svlcdaeval ivfstrgrly eyasnsvrst idrykkacsd vsntgsvsea ntqfyqqesn klrrqikdiqnsnrhilgea lsaltfkevk nletklekai srirskknem lfaeiehmqk riaeneraqeqmnlmpsshd hqyqtmgags qaqqqqpqsy darnflpvnl lepnhhysrh dqpplqlv

>Rhsim12G0003300
MERRCTGKGNGGEMGRGKIEIKRIENNTNRQVTFCKRRNGLLKKAYELSVLCEAEVALIV
FSSRGRVYEFANNSKALRTLFDWLIIMLSDMEGNGGEMGRGKIEIKRIENNTNRQVTFCKR
RNGLLKKAYELSVLCEAEVALIVFSSRGRVYEFANNSNKSTIERYKKAHSDTSNPCPTPEIN
ARFYQQESEKLRQQIQMLQNTSRNIMGEGLGSLNMKELKQLENRLERGITRIRSKKIAENE
RLQELSMVSGQEYNAIQAYLARNAHALQLNIMEDGTGPDHTAYPPHLPTDKKHSLHLG

>AT3G54340
margkiqikr ienqnrqvt yskrrnglfk kaheltvlcd arvsiimfss snklheyisntttkeivdl yqtisdvdvw atqyermqet krklletnrn lrtqikqrlg ecldeldiqelrrledemen tfklvrerkf kslgnqiett kkknksqqdi qknlihelel raedphyglvdnggdydsvl gyqiegsray alrfhqnhhh yypnhglhap sasdiitfhl le

>FaAG
MAYENKPNTDLDADAQRRLGRGKIEIKRIENTTNRQVTFCKRRNGLLKKAYELSVLCDAE
VALIVFSNRGRLYEYSNNSSVRETIERYKKACADTSTNGSASEATTQYYQQEAAKLHNQIN
ALQNINRGYMAEGLSNKNIKELKGMERKLERAITRIRSKKNELLFAEIEYMQKRELDLHN
NNQLLRAKIAENERQQQSIIAITGGHGSYEIVQPTQPFHEARNYFQVNALQPNIHQYSCHD
QVSLQLV

>Md15
MAYESKSLSLDSPQRKLGRGKIEIKRIENTTNRQVTFCKRRNGLLKKAYELSVLCDAEVAL
IVFSNRGRLYEYANNSVKGTIERYKKASADSSNTGSVSEASTQYYQQEAAKLRARIVKLQ
NDNRNMMGDALNSMSVKDLKSLENKLEKAISRIRSKKNELLFAEIEYMQKRELDLHNNN
QLLRAKIAENERASRTLNVMAGGGTSSYDILQSQPYDSRNYFQVNALQPNHQYNPRHDQI

SLQLV
